# Optimization of inpatient medication administration among persons with Parkinson’s disease: recommendations on pharmacy technology and workflow

**DOI:** 10.3389/fphar.2023.1254757

**Published:** 2023-11-14

**Authors:** Jeryl Ritzi T. Yu, Brent S. Sokola, Benjamin L. Walter

**Affiliations:** ^1^ St. Luke’s Medical Center, Institute for Neurosciences, Quezon City, Philippines; ^2^ University of the East Ramon Magsaysay Memorial Medical Center, Quezon City, Philippines; ^3^ Department of Pharmacy, Cleveland Clinic, Cleveland, OH, United States; ^4^ Center for Neurological Restoration, Department of Neurology, Neurological Institute, Cleveland Clinic, Cleveland, OH, United States

**Keywords:** Parkinson’s disease, inpatient, medication administration, pharmacy, recommendations

## Abstract

Individuals with Parkinson’s disease (PD) are vulnerable during hospitalizations due to the underlying complexities o1f symptoms, and acute illness or medication changes often lead to decompensation. Complications during hospitalizations are often due to worsening motor and nonmotor symptoms and commonly result from inaccurate medication regimens. Although the accuracy of medication administration relies on an interplay of factors, including patient status, transitions of care, coordination between the hospital prescriber and outpatient neurologist, etc., hospital pharmacists play an integral role in pharmacotherapy. The main aspects of pharmacy strategies aim to achieve timely administration of levodopa-containing medications, reduction of substitution and omissions of antiparkinsonian medications, and avoidance of antidopaminergic medications. This paper highlights critical areas for improvement and recommendations to minimize the impact of other factors from the pharmacy standpoint.

## 1 Introduction

Ensuring the accuracy of medication regimens is paramount in optimizing inpatient care for people with Parkinson’s disease (PWP). Inaccurate medication regimens and administration of contraindicated medications are associated with complications and poor outcomes (Martinez-Ramirez et al., 2015; Lertxundi et al., 2017; Yu et al., 2023). With increasing complications such as aspiration due to swallowing problems and falls due to worsened mobility from dyskinesias or orthostasis, which renders them unable to participate in physical therapy, the overall length of stay is prolonged (Martinez-Ramirez et al., 2015). Alternatively, the involvement of non-motor symptoms can result in the administration of contraindicated medications, further worsening motor function. For these reasons, it can be argued that managing PD medication regimen is the cornerstone of inpatient care. Further, the role of pharmacists in establishing a systematic approach to delivering PD-specific care is well recognized (Azmi et al., 2019).

Several factors influence the accuracy of medication administration, including awareness of the time-critical nature of PD medications, medication availability, nothing per orem (NPO) status, patient to nurse ratios. Nursing staff reported that administration time constraints had compelled them to take shortcuts such as removing medications from storage locations well before administration time or gathering more than one patient’s medications at a time, etc., all of which increase errors, hence, calling for a revision to the 30-minute rule (Institute for Safe Medication Practices, 2023). However, revision of the time constraint is not feasible for PWP as levodopa is time critical. In this paper, we highlight key areas utilizing pharmacy workflow and technology to minimize the impact of other factors contributing to inaccuracies in medication administration.

## 2 Improving the timely administration of time-critical Parkinson’s medications

### 2.1 Categorize levodopa products as “time critical”

Levodopa, the most efficacious medication for PD for over 50 years (Cotzias et al., 1967), has a short half-life, requiring multiple times a day dosing (Nutt, 2008). Accuracy of medication administration times in the hospital is critical as this population is vulnerable to deterioration of symptoms with dosing and timing deviations. Studies have highlighted challenges in adherence to medication administration schedules (Hou et al., 2012; Richard et al., 2022) when comparing administration times to inpatient orders. In a recent study, we examined the deviations between the hospital administration times and the patient’s outpatient regimen. We found that 47% had average hospital dose timing intervals that differed from outpatient timing intervals by greater than 30 min, which were associated with a longer length of stay (Yu et al., 2023).

The Centers for Medicare and Medicaid Services (CMS) defines time-critical medications as “those for which an early or late administration of greater than 30 minutes might cause harm or have a significant, negative impact on the intended therapeutic or pharmacological effect” (Department of Health & Human Services Centers for Medicare & Medicaid Services). Longer dosing intervals or delayed doses may result in problems related to wearing off, such as worsening of motor symptoms (tremor, dystonia, dysphagia, freezing of gait, falls) and nonmotor symptoms (anxiety, shortness of breath). On the other hand, dosing too early may result in dyskinesias which limit effective rehabilitation, psychosis, orthostasis and syncope (Aminoff et al., 2011a; Nance et al., 2020).

Administration of levodopa-containing products is time-critical and unique from one patient to another. In accordance with the CMS definition above, we recommend that hospitals label levodopa-containing products as time critical. The Institute for Safe Medication Practices (ISMP) and CMS recommend that time-critical medications be given within 30 min before or after their scheduled due time. Further, the CMS standard for the preparation and administration of drugs states that hospital policies and procedures should facilitate administration within this 1-hour time window. The timing of levodopa administration should be documented, including reasons for doses outside the recommended time window.

Special instructions must be in place to avoid missing doses in cases where the patient is off the floor. For short duration procedures and when time-critical medications are due within 15 min, it may be best to give the dose prior to wheeling patients off the floor. For longer procedures (ex. surgeries, etc.), time-critical medications must be given as soon as patients are transferred to the recovery room. Remaining doses of the day are to be timed based on the patient’s home dosing interval. Resume scheduled due times the following day based on the patient’s home regimen.

### 2.2 Emphasize the role of pharmacists in medication reconciliation

Deviation from the home regimen can be due to various shortfalls represented by the *Swiss cheese model* in which multiple gaps align—from order entry to bedside administration–resulting in a medication error reaching the patient (Reaso, 2000). On order entry, prescribers may enter the order based on outdated home medication lists, or the electronic medical record (EMR) may implement default administration times. Pharmacist review of medication orders is integral in double-checking the continuity of dose, route, frequency, and timing of PD medications from home regimens as required by Joint Commission Standard for Medication Management MM.05.01.01. Yet only 43% of hospitals have 24/7 pharmacy staffing with medication orders reviewed in real-time, and 7.5% have no method for prospective order review after hours requiring retrospective review after the pharmacy opens (Pedersen et al., 2021). Hospitals should maintain adequate staffing for prospective order review by pharmacists and ensure that the reviewing pharmacist has access to sufficient information to confirm the outpatient regimens. This includes EMR access to outpatient office visit notes, telephone notes, and outpatient pharmacy dispensing records.

### 2.3 Employ pharmacists to direct and indirect patient care roles

When feasible, hospital systems should employ pharmacists to fulfill specific roles. Aside from order review, pharmacy consultations on admission may also be considered to assist PWPs in establishing medication dose and timing. Whenever feasible, hospitals should staff decentralized floor-based pharmacists who can speak directly with patients, care partners, and the patient’s neurologist to establish an accurate account of home PD medication regimens. A helpful resource for the reconciliation of medication regimens is patient safety kits from the outpatient setting, such as the Parkinson’s Foundation Aware in Care kit (Parkinson, 2023). They contain medication forms and medical alert cards that may assist pharmacists with continuing the PWP’s outpatient regimen accurately while hospitalized. Floor-based pharmacists can also round with medical teams and assist nursing staff with impromptu medical problems. Hospitals should consider active interventions via PD consultation services with a physician, mid-level provider, pharmacist, and nurse trained in PD care. This serves as a resource for concerns surrounding special circumstances other staff may be unfamiliar with.

In addition to direct patient care roles, hospitals should staff pharmacists who provide institutional support through pharmacy informatics, formulary management, and review of medication safety events. Informatics pharmacists can contribute significantly by improving EMR alerts, pharmacy workflows, order sets, and order mapping to product selection. In addition, they play a role in ensuring that the automated dispensing cabinets (ADC) footprint is optimized for PD medications. Lastly, hospitals should staff pharmacists who are dedicated to formulary management and work with the hospital Pharmacy & Therapeutics committee to ensure all PD medications are on the formulary and available whenever feasible. Medication safety pharmacists collect and trend medication errors for the institution to identify opportunities for workflow or structural changes to prevent future events.

### 2.4 Consider internal pharmacy technician training programs

Recent pharmacy technician shortages have caused problems in hospitals nationwide. In a recent survey of hospital pharmacies, 73% reported technician staffing shortages, with an average vacancy rate for FTE positions of 13% (Schneider et al., 2022). This shortage has been detrimental to the multitude of functions pharmacy technicians support in our hospitals, including timely fulfillment of medication orders from centralized pharmacies, restocking of ADCs, controlled substance system management, inventory and purchasing, sterile compounding, billing and reimbursement, and re-dispensing medications from nursing or provider requests. A pharmacy technician shortage in these areas has spread staffing thin, making the essential functions of medication procurement and order fulfillment another barrier to the timely administration of medications. Hospitals can implement internal pharmacy technician training programs to promote internal relief from staffing shortages (Pereda et al., 2022).

### 2.5 Enforce custom order timing entries

Beyond staffing, several pharmacy informatics solutions may assist with accurate order entry. A common error is when the EMR incorrectly transcribes a PWP’s specific medication times and intervals. For example, a patient may take carbidopa/levodopa 25/100 mg, one tablet upon waking up, then every 4 hours daily (ex. 8 a.m., 12 noon, 4 p.m., 8 p.m.). The EMR may translate this to a hospital schedule “four times per day” (i.e., 6 a.m., 12 noon, 6 p.m., 12 a.m.), significantly deviating from home use with longer dosing intervals. Problems related to wearing off, such as mobility problems, falls, worsening of tremor, dystonia, dysphagia, freezing of gait, and other nonmotor symptoms, including shortness of breath and anxiety, may arise. Similarly, high protein meals can reduce absorption of levodopa and providers should request meal delivery 60 min after the nearest levodopa administration time, if hospital meal services can accommodate. On the other hand, dosing too early may cause dyskinesias, predisposing patients to falls, inability to participate in therapy, psychosis, orthostasis, and syncope (Aminoff et al., 2011a; Nance et al., 2020). Pharmacy informatics teams should consider removing default administration times for carbidopa-levodopa products on order entry in the EMR and instead force custom hour/minute time orders.

### 2.6 Utilize EMR overdue alerts

Hospitals may also leverage their EMRs to promote timely administration of levodopa products. For instance, most nursing medication administration records (MARs) have overdue alerts. However, most hospitals permit medications to be given within 1 h of the due time before they are considered overdue. Hospitals should update their MARs to reflect the time-critical window of 30 min, and the MAR should flag nurses of overdue PD medications accordingly. Additionally, hospitals should include carbidopa-levodopa as a time-critical medication in their nursing onboarding and through continuing education. This education can be reinforced by working with pharmacy informatics teams to add administration comments to medication files stating that the medication should be given within 30 min of its due time.

### 2.7 Optimize dispensing through automated dispensing cabinets (ADCs) and labeling

There are other systems hospitals can implement to reduce deviations. First, the medication must be available to the nurse when due. Most hospitals utilize a mix of order fulfillment from a centralized pharmacy delivery and ADCs on patient floors. In a survey, 75% of hospitals now use ADCs as the primary dispensing method for maintenance medications (Pedersen et al., 2021). Although space is limited in ADCs, and hospital pharmacies must judiciously choose which meds are stocked, we recommend, at a minimum, that immediate-release carbidopa-levodopa be stocked on each floor to minimize delays. For PD medications not able to be stocked in ADCs, hospital pharmacies should ensure their cartfill deliveries dispense at least a 24 h supply of a medication order. Multiple deliveries for the same medication order throughout the day require more pharmacy staffing resources and introduce more opportunities for the late arrival of time-critical medications. Using a charge-on-administration model, rather than a charge-on-dispense model, ensures patients are not charged for supplies sent by the pharmacy and unused. Another strategy to optimize medication dispensing is through barcode labeling. Practices vary across different hospitals. For example, larger hospitals are more likely to utilize barcode scanning to verify ingredients during intravenous medication compounding (Pedersen et al., 2021). Medications can be labeled through an electronic health record-integrated mobile dispense tracking to reduce redispense rates (Bhakta et al., 2022), thereby increasing administration efficiency by reducing redispense associated delays.

### 2.8 Establish standard protocols for event reporting and data analysis

Standard procedures should be in place if delays in the administration of levodopa-containing medications occur. These include prescriber notification to evaluate the need to change the timing of future levodopa doses and monitoring of any change in motor or nonmotor symptoms (Code of Federal, 2012). Typical scenarios include transitions of care, such as the emergency department and post-anesthesia care unit, which are susceptible to dose omissions and delays. Regardless of intentionality (for example, when patients are tested in the off-mediation state during deep brain stimulation surgery), a standard process for reporting untimely administration of levodopa-containing medications should be established and implemented. For levodopa-containing medications, nursing staff should reference the MARs to establish succeeding dose and timing intervals if the previous dose was off schedule. In addition, medication safety pharmacists can assist with reviewing reported levodopa administration events or coordinate reviews with other area-specific pharmacists. Recommendations are summarized in Table 1.

**TABLE 1:** Modified from ISMP Acute Care Guidelines for Timely Administration of Scheduled Medications. Adapted for time-critical Parkinson’s medications^a^.

Topic	Description
Maintain Adequate Staffing Levels	• Maintain adequate nursing staffing to allow the administration of time-critical medications that may not align with standard administration times
	• Maintain adequate pharmacist and pharmacy technician staffing to allow for prospective order review and timely dispensing of medications
Use of automated Dispensing Cabinets (ADCs)	• Ensure the number of cabinets allows for the timely removal of medications
	• Stock levodopa products in at least one ADC per floor whenever possible
	• Limit overrides to emergency situations
Justification of early or late administration	• Define justifiable reasons that levodopa medications may be given early or late
	• Require notification of provider to guide possible adjustment of future scheduled doses
MAR documentation	• Require nursing staff to document the exact time the drug was administered in the MAR and provide documentation of reasoning for doses of levodopa-containing products given outside the recommended 1-h window for critical medications
Reference MARs	• Require nursing staff to reference the MAR showing times of previously administered dose to assess if dosing interval is off schedule
eMAR alerts	• Enable eMAR alerts to show when doses of levodopa-containing medications are soon to be overdue (30 min)
	• Highlight previous doses on the eMAR that have been given late or omitted
	• Enable eMAR alerts to alert staff who administer medications when attempting to administer contraindicated medications and document rationale if overridden
Standard Administration Times	• Levodopa medications should be classified as time-critical and custom times (i.e., specific times) should be entered for accuracy of administration times
	• Excluding time-critical medications, utilize standard administration schedules whenever possible to reduce the time burden on staff who administer medications
Procedure to follow if medication administration is early or delayed	• Establish a procedure for staff to follow if levodopa-containing medications will be or have been delayed. This should include prescriber notification to evaluate the need to change the timing of future doses and monitor for any change in clinical condition. Medication errors due to late/missed doses should be reported to the attending physician
Event reporting	• Establish a process for reporting untimely administration of levodopa-containing medications, even if the reason for the delay is justifiable
Data analysis	• Employ dedicated medication safety pharmacists who can assist with reviewing reported events or coordinate reviews with other area-specific pharmacists. These reviews should not be punitive but used to develop system-based process improvement initiatives to prevent further untimely administration
Other Pharmacy Resources	• Employ medication safety pharmacist(s) to assist with medication event reporting and quality improvement initiatives
	• Employ pharmacy informatics pharmacist(s) and technician(s) to improve EMR alerts, workflows, order sets, order mapping to product selection, and ensure ADC footprint is optimized for PD medications
	• Employ pharmacist(s) dedicated to formulary management who can work with the hospital Pharmacy & Therapeutics committee to ensure all PD medications are on the formulary and available, whenever feasible
	• Employ floor-based pharmacists who directly confirm home medications with patients and care partners, reconcile inpatient orders, round with medical teams, and assist nursing staff with impromptu medication problems
	• Implement EMR alerts to appear for contraindicated medications on prescribing, pharmacist verification, and nurse administration
	• Implement EMR alerts to warn nursing staff when a levodopa-containing medication is overdue (<30 min)
	• Remove default EMR administration times for levodopa-containing medications and require prescriber entry of home administration times
	• Implement policies and procedures which empower pharmacists to reduce nursing workload by adjusting MAR due times for non-time critical medications
	• Utilize the EMR to guide prescribers on policies and procedures for using patient-owned supply or a product substitution that best approximates home LEDD if a non-formulary PD medication is ordered

^a^ Reprinted with permission, Copyright 2011, Institute for Safe Medication Practices. www.ismp.org. 5,200 Butler Pike, Plymouth Meeting, PA 19462. This material is protected by copyright laws and may not in whole, in part, or by reference be used in any advertising or promotional material, or to compete with ISMP.

## 3 Reducing omissions and substitutions of unavailable medications

Deviations from outpatient regimens may also occur through omissions and substitutions, resulting in differences in levodopa equivalent daily dose (LEDD). In our recent study, LEDD deviation occurred on 43% of days; 68% LEDD underdose where patients received a lower LEDD (median of 150 units less) in the hospital than in their outpatient regimen. Levodopa substitutions were identified in 19% of hospital days. The most common formulation substitutions were substituting an extended-release (ER) tablet of the same dose from the patient’s outpatient regimen for an immediate-release (IR).

### 3.1 Stock appropriate medications in the formulary for common hospital situations

Medication formulation availability contributes to dose omissions and substitutions (Lertxundi et al., 2017). Unfortunately, many hospitals’ formularies do not include all PD medications (Lertxundi Etxebarria et al., 2021). The FDA Orange Book defines therapeutic equivalency and specific drug products that can be confidently interchanged while assuming comparable safety and efficacy (US Department of Health and Human Services Food and Drug Administration, 2023). Many PD medications have specific formulations involving release mechanisms that are not therapeutically equivalent. Neurologists should work with their hospital pharmacy and therapeutics (P&T) committee to ensure the various levodopa formulations are added to the hospital formulary and stocked for inpatient use.

Aside from limitations from hospital logistics, medication omissions may occur due to NPO status. Carbidopa/levodopa is dosed several times daily due to its short half-life, resulting in problems when patients are placed on NPO status longer than necessary. A study on perioperative medication withholding found that the levodopa median withholding time was 12.35 h (Fagerlund et al., 2013), equivalent to 2-4 doses missed depending on patient profile. Some PWPs may also be placed on NPO due to nausea or swallowing difficulties, resulting in missed doses while awaiting formal evaluation by a speech-language pathologist (SLP). Hospitals should stock carbidopa-levodopa oral disintegrating tablets for use in such situations. These tablets dissolve before swallowing, and a PWP may still be able to take this when unable to swallow other formulations whole. Other alternatives such as rotigotine transdermal patch, inhaled levodopa, or apomorphine sublingual and subcutaneous injection are also options but limited in formularies (Lertxundi Etxebarria et al., 2021), likely due to cost. Additionally, these options may not be appropriate for many patients due to comorbidities or complications of PD.

### 3.2 Consider temporarily using the patient’s or nearby hospital supply when medications are unavailable

If formulary addition is not feasible, a simple and efficient process should be developed to have the inpatient pharmacy identify patient-owned supplies and re-labeled them for use during the hospital stay. In some situations, borrowing supplies from a nearby hospital in a hub-and-spoke model might be acceptable. Still, this practice is suboptimal since many PD medications are time critical. As a last resort, when levodopa medication is not on the hospital formulary, patient-owned supply is unavailable, and external supply is not quickly available from nearby institutions, an interchange resulting in the equal LEDD (Lertxundi et al., 2015) is preferable to omission.

### 3.3 Develop user-friendly interchange protocols

Although complete stocking of medications and hospital pharmacist review has improved medication administration (Nance et al., 2020; Lance et al., 2021) this may only be feasible in some hospitals. Furthermore, levodopa formulations are varied and complex, with different pharmacokinetic and pharmacodynamic properties by formulation (Nutt, 2008; Espay et al., 2017). An alternative therapeutic interchange protocol with equivalent levodopa immediate-release dose has been proposed (Tomlinson et al., 2010; Lertxundi et al., 2015). However, converting from a patient’s outpatient to a hospital regimen is still error-prone, especially among clinicians inexperienced with PD care (Grissinger, 2018). Neurologists should work with the P&T committee and pharmacy informatics to implement an interchange protocol using LEDD conversion guides between different levodopa formulations (Espay et al., 2017; Tomlinson et al., 2010; Schade et al., 2020a; Julien et al., 2021) and leverage the EMR to guide clinicians in selecting appropriate conversions.

In cases where patients are sedated or mechanically ventilated, early efforts should be made to obtain enteral access as soon as possible by methods such as nasogastric tubes. PD medications should be converted to LEDD (Schade et al., 2020a) and administered as crushed immediate release carbidopa-levodopa tablets in divided doses. Consultation with a PD specialist and/or pharmacist is highly recommended. Close monitoring for PD symptoms and dyskinesias is necessary and doses and/or intervals may need to be modified based on response.

## 4 Reducing administration of contraindicated medications to people with Parkinson’s

Due to the fundamental PD pathophysiology involving loss of dopaminergic cells, medications affecting dopaminergic states must be used cautiously. In particular, dopamine antagonists belonging to the antipsychotic (including haloperidol, fluphenazine, chlorpromazine, risperidone, olanzapine, ziprasidone, aripiprazole, etc.) and antiemetics drug classes (including metoclopramide, promethazine, prochlorperazine) have dopamine receptor blocking properties. These further worsen mobility which increases risk for falls, may exacerbate cognitive impairment, possibly increasing risk for psychosis, and swallowing, which increases risk for aspiration (Aminoff et al., 2011a; Gerlach et al., 2011; Ahlskog, 2014). These complications result in extended hospital stays and increased fatalities (Lertxundi et al., 2017; Grissinger, 2018).

Antidopaminergic medications are often given when patients decompensate. In our study, contraindicated medications were administered at least once in 10% of admissions, the top three of which were available in non-parenteral formulations (Yu et al., 2023). We found that 61% (88/143) and 20% (29/143) of haloperidol was given via intravenous and intramuscular routes, respectively, and only 18% (26/143) were given orally as a tablet. However, of those given olanzapine, 80% (76/95) was oral, while only 20% (19/95) was intramuscular, suggesting that factors other than tolerability may play a role in the administration of contraindicated medications.

In cases of acute agitation or psychosis during which patients pose a risk to themselves and others, antipsychotics with antidopaminergic medications (i.e., haloperidol, risperidone, olanzapine, etc.) are used. While this and other studies raise a concern about the safety of high-affinity D2 antagonists in people with PD, there is a paucity of controlled studies examining the comparative efficacy and safety of different algorithms for managing acute agitation and psychosis in PD. Controlled studies examining the ideal management of acute agitation and psychosis in PD are lacking. Current data suggests that one should minimize the use and potency of D2 antagonists in PD and use sound clinical judgment to escalate the potency of medications used only when absolutely necessary. Neurologists should encourage using technical and staffing resources to guide judicious use. We highlight strategies from the pharmacy standpoint to optimize medication selection.

### 4.1 Avoid ADC overrides

Acutely agitated patients may cause harm to themselves and others, and ADCs are often set up to allow for medication overrides in emergent situations. An override occurs when a medication order is placed, but the medication is removed before the pharmacist reviews the order. However, not all instances of agitation in the hospital warrant this level of urgency, and ISMP discourages the overuse of ADC overrides, as bypassing pharmacist review increases the risk of medication errors (Institute for Safe Medication Practices, 2019). Overriding the use of antipsychotics for acute agitation in PWP removes the opportunity for a pharmacist to intervene and recommend alternatives such as benzodiazepines. Neurologists should work with hospital rapid response and psychiatry teams to discourage the use of the override process whenever possible and educate on alternatives to antipsychotics for managing acute agitation in PWP. Nevertheless, challenges continue to exist in managing acute agitation among PWPs in the inpatient setting, which should be studied further.

### 4.2 Utilize EMR drug-disease interaction alerts

Other scenarios when contraindicated medications are commonly given include encephalopathic or uncooperative patients and those critically ill or unable to tolerate oral feeding. When necessary, medications that have less extrapyramidal side effects such as quetiapine, clozapine or pimavanserin, are ideal. However, these medications are only available per orem and would not be favorable in scenarios when patients are unable to tolerate oral feeding. In these situations, providers sometimes prefer medications with intravenous or intramuscular formulations, even if they have extrapyramidal side effects. ADC overrides should be avoided so as to allow pharmacist review (Institute for Safe Medication Practices, 2019). Medication safety pharmacists and pharmacy informaticists can assist with clinical decision support for drug-disease contraindications with recommended dose ranges and frequencies. Specifically, EMR alerts can be formulated to remind prescribers on order entry, pharmacists on prospective order review, and nurses on administration that these medications are contraindicated in patients with PD. The American Parkinson Disease Association page contains a list of medications to avoid or use cautiously among PWPs (American Parkinson Disease Association, 2018).

### 4.3 Exclude antidopaminergic medications in standard order sets

Certain institutions utilize a standard postoperative order set intended for the general patient population. In addition to EMR alerts, teams that frequently see PWP should consider removing these contraindicated medications with dopamine antagonist properties from shared admission order sets and standard peri-operative order sets. This is crucial in perioperative situations such as perioperative nausea when a patient is commonly placed on NPO or given medications containing antidopaminergic properties, worsening motor function. By removing contraindicated medications in the standard order sets, the provider is forced to order the medication, which is subject to EMR alerts, etc. A better alternative is to replace contraindicated medications with PD-safe medications for common scenarios encountered. For example, consider revising standard order sets to indicate the use of ondansetron over metoclopramide, promethazine, and prochlorperazine for antiemetics among PWPs, and removing haloperidol from order sets for those experiencing agitation. Recommendations are summarized in Table 1.

## 5 Conclusion

In summary, the challenges surrounding the accuracy of administering time-critical PD medications are well recognized. Approaches from pharmacy technology, staffing, and workflow can be utilized to minimize the impact of other factors contributing to inaccuracies in medication administration. Proactive interventions that utilize pharmacists as part of a multifaceted approach are integral in ensuring a safe hospitalization for PWPs.
